# Efficacy and safety of preoperative intravenous iron versus standard care in colorectal cancer patients with iron deficiency anemia: a systematic review and meta-analysis

**DOI:** 10.1097/MS9.0000000000002727

**Published:** 2024-11-11

**Authors:** Pishoy Sydhom, Mahmoud Shaaban Abdelgalil, Bakr Al-Quraishi, Nahla Shehata, Mohamad El-Shawaf, Nourhan Naji, Nouran Awwad, Mohamed Tarek Osman, Abdelmonem Mahmoud, Ahmed K. Awad

**Affiliations:** a Department of General Surgery, Ain-Shams University Hospitals, Cairo, Egypt; b Faculty of Medicine, Ain-Shams University, Cairo, Egypt

**Keywords:** colorectal cancer, intravenous iron, iron deficiency anemia, meta-analysis, standard care, systematic review

## Abstract

**Background:**

Anemia, particularly iron deficiency (ID) anemia, is common in colorectal cancer (CRC) patients, affecting up to 58% of individuals. This study aimed to compare the effectiveness and safety of preoperative intravenous iron (IVI) with standard care (no iron or oral iron) in CRC patients with ID anemia.

**Methods:**

A systematic search across multiple databases identified studies comparing IVI versus no iron or oral iron in CRC patients with ID anemia. Pooled data were analyzed for changes in hemoglobin (Hb) levels, need for red blood cell transfusions (RBCT), overall mean number of transfused RBC units, overall survival (OS), disease-free survival (DFS), and complications.

**Results:**

The authors analyzed data from 11 studies with 2024 patients and found that IVI significantly increased Hb levels at crucial time points: preoperative (MD=1.17, 95% CI [0.95–1.40], *P*<0.01), postoperative day one (MD=1.32, 95% CI [0.89–1.76], *P*<0.01), hospital discharge (MD=0.76, 95% CI [0.28–1.24], *P*=0.002), and 30 days postoperative (MD=1.57, 95% CI [1.27–1.87], *P*<0.01). IVI significantly decreased the overall need for RBCT, particularly in the postoperative period (RR=0.69, 95% CI [0.52–0.92], *P*=0.01). It also reduced the mean number of transfused RBC units, total complications, and wound dehiscence. However, there were no significant differences in total death, hospital stay, infections, paralytic ileus, OS, or DFS.

**Conclusion:**

Preoperative IVI significantly increased Hb levels at critical time points and markedly reduced the overall need for RBCT, complications, and wound dehiscence. To further validate these findings and ensure robust conclusions, more well-designed randomized controlled trials are warranted.

## Introduction

Highlights

- Anemia, particularly iron deficiency anemia, is common in colorectal cancer patients.
- This study compared preoperative intravenous iron (IVI) with standard care.
- IVI significantly increased hemoglobin levels at crucial preoperative and postoperative time points.
- IVI reduced the need for red blood cell transfusions, especially postoperatively.
- IVI decreased total complications and wound dehiscence compared to standard care.
- No significant differences were found in mortality, hospital stay, or survival outcomes.
- More well-designed randomized controlled trials are needed to validate these findings.

Colorectal cancer (CRC) stands as a significant global health concern, ranking as the second most common cancer in women and the third most common in men^1–3^. In 2020 alone, it affected over 1.9 million people, with projections estimating a substantial increase to 3.2 million new cases and 1.6 million deaths annually by 2040^1^. With anemia is a common complication in CRC patients affecting up to 58% of cases^4,5^, iron deficiency (ID) anemia is the most prevalent, resulting from chronic tumor-induced blood loss, poor nutrition, or gastrointestinal malabsorption. Notably, the connection between ID and heightened oncogenicity emphasizes the critical role of iron in maintaining immune functions. Furthermore, anemia in CRC patients is associated with reduced overall survival^6–8^.

Prior research indicates that preoperative anemic CRC patients are more likely to receive allogeneic red blood cell transfusions (RBCT)^9^. The overall transfusion rate for CRC patients is 23.8%, with the highest frequency occurring intraoperatively and postoperatively^5^. Allogeneic RBCT is, in turn, linked to elevated risks of all-cause mortality, postoperative infections, surgical reintervention, and a poorer oncological outcome, including cancer recurrence^10–15^. Given surgery’s impact on immune function, minimizing risks from perioperative RBCT is crucial, as observational data reveal a correlation between the number of units of blood administered and complication rates following major gastrointestinal and noncardiac surgeries^16,17^. With early recognition and management of pre-existing anemia is crucial for patient blood management, thus preoperative Intravenous iron (IVI) supplementation has been suggested as an approach to reduce the requirement for RBCT in CRC patients^12,15^.

Conflicting evidence exists regarding the effects of IVI on RBCT. Cohort studies have demonstrated a significant rise in Hb levels and lower transfusion rates when treating anemic patients with preoperative IVI^18–22^. In contrast, a systematic review suggested that IVI primarily increases Hb without reducing the rate of allogeneic blood transfusion^21^. To address this conflicting evidence, we aimed to conduct a systematic review and meta-analysis to compare the safety and efficacy of preoperative IVI versus standard care (oral iron or no iron) in CRC patients with ID anemia.

## Methods

The conduction of this systematic review and meta-analysis adhered to the 2020 Preferred Reporting Guidelines for Systematic Reviews and Meta-Analyses (PRISMA) (Supplemental Digital Content 1, http://links.lww.com/MS9/A637)^23^ and was guided by the recommendations in the Cochrane Handbook of Systematic Reviews of Interventions^24^. The work has been reported in line with the Assessing the Methodological Quality of Systematic Reviews (AMSTAR) (Supplemental Digital Content 2, http://links.lww.com/MS9/A638) guidelines. A comprehensive protocol was registered with the International Prospective Register of Systematic Reviews (PROSPERO).

### Search strategy

A comprehensive literature review was conducted using PubMed, Web of Science, Scopus, and Cochrane Central Register of Controlled Trials (CENTRAL) from inception to 20 September 2023. The search strategy was related to medical terms, such as intravenous iron, preoperative anemia, and colorectal cancer. We have provided the full search strategy in the Supplementary Table 1 (Supplemental Digital Content 3, http://links.lww.com/MS9/A639).

### Eligibility criteria and studies selection

Two authors screened the search results to assess the eligibility of the articles for our study, concentrating on randomized controlled trials (RCTs), nonrandomized comparative studies, and observational studies (both prospective and retrospective cohorts). The initial screening involved titles and abstracts followed by a detailed review of the selected study texts.

We included studies that compared IVI to the standard of care, which could be oral iron or no iron, for the treatment of preoperative anemia in colorectal cancer patients. The inclusion criteria were patients aged 18 years or older diagnosed with colon cancer, and ID anemia was defined using the WHO criteria: Hb <13 g/dl in men, Hb <12 g/dl in women, serum ferritin <30 ng/ml, and transferrin saturation index <20%.

In our analysis, the primary outcomes focused on changes in mean hemoglobin (Hb) levels, the need for red blood cell (RBC) transfusions, and mean units of RBC transfusions. The secondary outcomes included hospital stay, total deaths, all complications, total infections, wound infections, wound dehiscence, paralytic ileus, overall survival, and disease-free survival. Our comprehensive analysis aimed to provide a thorough understanding of the efficacy and safety of this treatment in surgical settings. Disparities were resolved through consultation with the senior author.

### Exclusion criteria

We excluded non-English publications, studies with unclear or insufficient outcome reporting, those focusing on pediatric populations, and those that did not address preoperative anemia in colorectal cancer. Studies with interventions other than IVI or standard care as well as those not directly comparing IVI with standard care were also excluded. Our criteria ensured the exclusion of studies focusing on conditions other than colorectal cancer and those lacking key outcome reporting. Case reports, editorials, reviews, and conference abstracts were omitted to maintain data robustness in our systematic review and meta-analysis.

### Quality assessment

We used the Cochrane risk of bias 2 (Rob2) tool^25^ to assess the quality of the randomized clinical trials and the Newcastle–Ottawa Scale (NOS)^26^ to assess the quality of the included observational studies. Two independent authors (A.M. and M.W.) evaluated the quality of the studies and discrepancies were resolved through discussion.

### Data extraction and study outcomes

Two independent authors systematically extracted pertinent data from each included study using a standardized Excel sheet. Discrepancies were resolved through discussion. The extracted data encompassed various aspects, including the studies’ characteristics such as country, type of IVI, dose of IVI, dose of oral iron, sample size of each group, inclusion and exclusion criteria, patients’ baseline characteristics such as males, BMI, ASA score, diabetes, hypertension, heart disease, previous stroke, iron treatment at diagnosis, tumor size, baseline Hb, baseline ferritin, and baseline transferrin saturation, and the outcomes of interest.

### Outcome definitions

Overall survival is the duration measured from the initiation of treatment to either the occurrence of death or the latest follow-up. Additionally, disease-free survival is the period after completing treatment during which the patient remains free of symptoms and signs of the disease.

### Data synthesis and assessment of heterogeneity

Statistical analysis was conducted using Revman software Version 5.4.1. The combined risk ratio (RR) for dichotomous data and the mean difference (MD) with 95% CI for continuous data were calculated. Statistical significance was indicated by a *P*-value below 0.05, while heterogeneity was assessed with a *P*-value less than 0.10, leading to the application of a random-effects model in its presence. Furthermore, a leave-one-out test was performed to identify the study accountable for any observed heterogeneity. Assessing publication bias was performed whenever an outcome had at least 10 studies^27^.

## Results

### Literature search results

**Figure 1:**
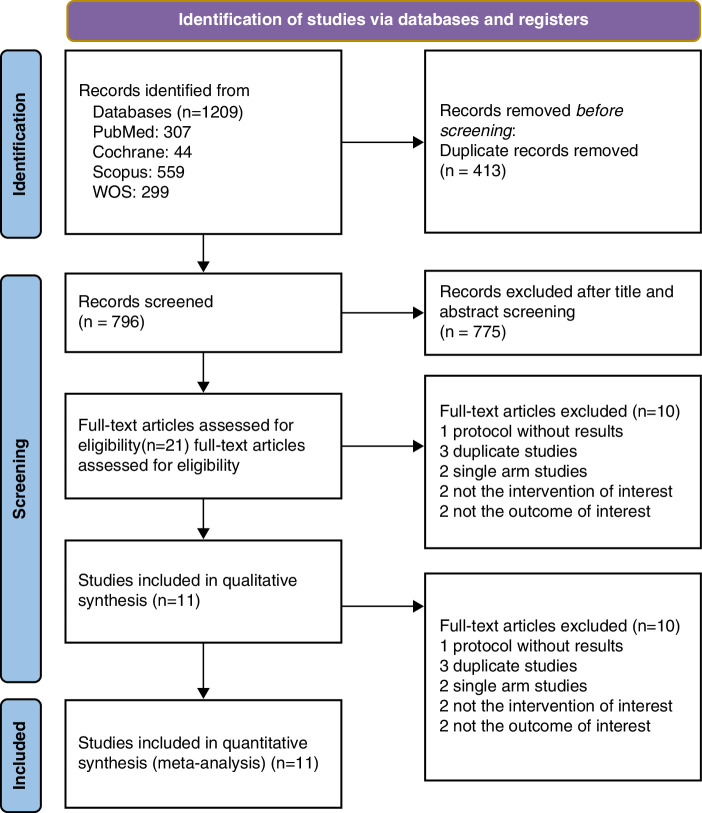
Prisma flow diagram.

Applying our search strategy to different databases yielded a total of 1209 studies of which 413 studies were duplicates and excluded. Subsequent title and abstract screening excluded 775 studies, leaving 21 studies for full-text screening. Following a detailed assessment, 10 studies were excluded, resulting in the inclusion of 11 studies in the final analysis^15,20,22,28–35^ Among these, five were RCTs^15,28,32,33,35^, and six studies^20,22,29–31,34^ were observational. The reasons for exclusion are outlined in the PRISMA flowchart diagram in Figure 1.

### Characteristics of individual studies

Our meta-analysis incorporated a total of 2024 participants from 11 studies, with 1044 in the IVI arm and 980 in the standard care arm. Approximately 45% of participants were female. Baseline Hb levels varied from 8.43 to 12.0 g/dl, reflecting differences in anemia severity and eligibility criteria across studies. The choice of IVI varied, with ferric carboxymaltose being the most common. The dose of IVI therapy ranged from 500 mg to 2000 mg, contingent on the type of intravenous iron, body weight, and anemia severity. Details for each included study are summarized in Table 1, while baseline characteristics are outlined in Table 2.

**Table 1:** Comprehensive overview of the included studies.

Study ID	Study design, country, study duration	Sample size of each group	Inclusion criteria	Indication for transfusion	Type of IVI	Dose of IVI	Type of standard care	Dose of oral iron	Conclusion
Edwards *et al*., 2009^32^	Randomized prospective blinded placebo-controlled trial, UK, 27 months	IVI= 34 Placebo= 26	Patients scheduled to undergo a bowel resection procedure for suspected colorectal cancer. Inclusion criteria involved individuals who had received a blood transfusion recently or had surgery scheduled within 15 days of the recruitment date	NR	Iron sucrose	600 mg in two divided doses	IV placebo 0.9 saline	NA	The study did not find any evidence to support the use of intravenous iron sucrose as a preoperative supplement to raise preoperative hemoglobin levels, reduce the need for allogeneic blood transfusion, or shorten hospitalization duration in patients undergoing colorectal cancer resection surgery
Calleja *et al*., 2016^34^	Noninterventional study prospective, Spain, 7 months	IVI= 111 No IVI= 155	Patients aged 18 or older diagnosed with colon adenocarcinoma located at least 15 cm from the anal margin who were scheduled for elective curative surgery. Iron deficiency anemia was defined using WHO criteria: Hemoglobin (Hb) <13 g/dl in men, Hb <12 g/dl in women, serum ferritin <30 ng/ml, and transferrin saturation index <20%. The study did not restrict the surgical methods used, allowing for laparoscopy, open surgery, single port, and other approaches	RBC transfusions were guided by blood tests and hospital protocols: Required for hemoglobin levels <7 g/dl. Administered based on physician criteria for levels between 7 and 9 g/dl. Not recommended for levels >9 g/dl	Ferric carboxymaltose	Median dose 1000 mg	No-IV iron	NA	Administering preoperative ferric carboxymaltose to colorectal cancer patients with iron deficiency anemia had several positive outcomes: There was a significant reduction in the need for red blood cell transfusions. Patients had shorter hospital stays. The treatment led to higher rates of positive response. A greater percentage of patients achieved normal hemoglobin levels both upon admission to the hospital and 30 days after surgery
Keeler *et al*., 2017^15^	RCT, UK, 25 months	IVI= 55 Oral= 61	Patients with anemia and non-metastatic colorectal adenocarcinoma were included in the study. Enrollment occurred at least 2 weeks before their scheduled surgery. The inclusion criteria for hemoglobin levels were set at 1 g/dl lower than the WHO definition, requiring levels below 11 g/dl for women and below 12 g/dl for men	Committee guidelines recommend considering transfusion if hemoglobin levels are <8.0 g/dl. Transfusion is typically indicated for levels <7.0 g/dl. The decision to transfuse should depend on the patient’s clinical condition	Ferric carboxymaltose	A maximum dose of 1000 mg was administered per week and a maximum of 2000 mg during the trial	Oral ferrous sulphate iron	200 mg twice daily	Intravenous (IV) iron did not reduce the need for blood transfusions in patients undergoing colorectal cancer surgery. However, IV iron was more effective than oral iron in addressing preoperative anemia and iron deficiency in these patients
Laso-Morales*et al*., 2017^20^	Retrospective cohort study, Spain, 23 months	IVI= 232 Standard care= 90 (67 no iron, 23 oral iron)	The study included patients with anemia. These patients were scheduled for elective surgery to remove colorectal cancer. Preoperative anemia was defined as having hemoglobin (Hb) levels below 13 g/dl for both males and females	Most patients followed a restrictive transfusion trigger of Hb <8 g/dl. A less restrictive trigger of Hb <9 g/dl was used for patients with active cardiac disease or acute anemia symptoms. This transfusion protocol was consistently applied by anesthesiologists and surgeons throughout the hospital stay, including in the operating theater, anesthesia recovery unit, and wards	Iron sucrose, Ferric carboxymaltose	Iron sucrose 200 mg up to three times a week preoperatively. Ferric carboxymaltose 500– 1000 mg once a week preoperatively	Standard care (oral iron or no iron)	100 mg	Intravenous iron (IVI) was more effective than standard care in managing preoperative anemia in colorectal cancer patients. IVI appeared to reduce infection rates. However, it did not lead to a decrease in the need for postoperative red blood cell transfusions when compared to standard care
Wilson *et al*., 2018^22^	Retrospective cohort study, Netherlands, 78 months	IVI= 102 No IVI= 218	The study included all individuals who underwent colorectal cancer resection surgery at the Department of Surgery, Reinier de Graaf Hospital, the Netherlands, between 1 January 2010, and 1 July 2016. Eligible participants were patients diagnosed with anemia, defined as hemoglobin levels below 8.0 mmol/l (12.9 g/dl) for men and below 7.5 mmol/l (12.0 g/dl) for women	NR	Iron (III) carboxymaltose, iron (III) isomaltoside	1000–2000 mg	No-IV iron	NA	The study examined the effects of 1000–2000 mg of preoperative intravenous iron therapy. It found that this treatment did not have a significant impact on the long-term overall and disease-free survival of colorectal cancer patients with anemia
Kam *et al*., 2020^31^	Cohort study, Hong Kong, 19 months	IVI= 38 No IVI= 62	The study included individuals diagnosed with colorectal adenocarcinoma. Eligible participants were identified as having preoperative iron deficiency anemia, with hemoglobin levels below 10 g/dl before receiving a blood transfusion or below 12 g/dl after a recent transfusion	Admission hemoglobin levels ≤8 g/dl typically necessitated a transfusion. The decision was at the discretion of the surgeon or anesthetist, particularly during perioperative or postoperative phases	Iron sucrose, Iron isomaltoside	Iron sucrose 500 mg with a total of two doses Iron Isomaltoside 1000 mg (or 20 mg/kg if bodyweight is less than 50 kg)	No-IV iron	NA	Intravenous iron (IVI) can significantly increase hemoglobin levels. This treatment is effective for patients with iron-deficiency anemia before colorectal surgery. It results in a reduced need for red blood cell transfusions
Dickson *et al*., 2020^33^	RCT, UK, 22 months	IVI= 45 Oral= 65	The study included colorectal cancer patients with anemia who had chosen to undergo surgery. Anemia was defined as having a hemoglobin level 10 g/l or lower below the sex-specific WHO criteria, which were ≤120 g/l for women and ≤130 g/l for men	NR	Ferric carboxymaltose	Dosed by weight and hemoglobin in accordance with the summary of product characteristics	Oral ferrous sulphate iron	200 mg twice daily	Intravenous iron (IVI) is recommended for correcting preoperative anemia. The method of iron therapy administration did not significantly affect survival outcomes. Correcting preoperative anemia may lead to an overall survival benefit in patients undergoing elective colorectal cancer surgery
Kangaspunta *et al*., 2022^30^	Retrospective cohort study, Finland, 24 months	IVI= 180 No IVI= 138	The study included colon cancer patients with anemia. These patients underwent surgery at Helsinki University Hospital (Jorvi) during the years 2017–2018	NR	Ferric carboxymaltose	500 mg or 1000 mg	No-IV iron	NA	A significant reduction in postoperative complications in colon cancer patients with anemia. Who received preoperative intravenous iron supplementation therapy. The treatment also decreased the incidence of postoperative anemia with a quicker recovery for patients
Ploug *et al*., 2022^29^	Retrospective cohort study, Denmark, 81 months	IVI= 122 No IVI= 48	The study included patients undergoing surgery for colorectal cancer. Eligible patients had a diagnosis of iron deficiency anemia (IDA) at the time of their colorectal cancer diagnosis. Anemia was defined using WHO criteria, with hemoglobin levels below 13.0 g/dl (8.1 mmol/l) for males and below 12.0 g/dl (7.4 mmol/l) for females. Iron deficiency was defined as having a serum ferritin level below 50 mg/l	The guideline recommends RBC transfusion for: Hb <6.9 g/dl in patients without cardiac comorbidities. Hb <8.1 g/dl in patients with cardiac comorbidities (ischemic or valvular heart disease). Hb <9 g/dl in patients with recent acute myocardial infarction. Outside these limits, transfusion is only advised in the presence of specific anemia symptoms (angina, orthostatic hypotension, tachycardia)	Ferric derisomaltose, Iron isomaltoside	Single dose according to a modified Ganzoni formula. The maximal dose to be administered was 20 mg/kg. MEDIA*N*=1000	No-IV iron	NA	Administering intravenous iron treatment before surgery did not lead to increased hemoglobin levels during the surgery. It also did not reduce the likelihood of colorectal cancer (CRC) patients with iron deficiency anemia requiring perioperative red blood cell transfusions (RBCT)
Fung *et al*., 2022^35^	RCT, Hong Kong, 21 months	IVI= 20 No IVI= 20	The study included patients diagnosed with colorectal cancer scheduled for elective tumor resection surgery with an expected waiting period of at least 3weeks. Adults with anemia (defined as hemoglobin below 130 g/l) and iron deficiency (indicated by ferritin levels below 30 μg/l or in the range of 30 to 100 μg/l with transferrin saturation below 20%) were considered for participation	NR	Iron isomaltoside	20 mg.kg^-1^ up to 1000 mg	No-IV iron	NA	Iron isomaltoside therapy was safe and had a minor effect on hemoglobin level changes during the perioperative period
Talboom *et al*., 2023^28^	RCT, Netherlands and Italy, 76 months	IVI= 96 Oral= 106	The study included adult patients aged 18 years or older. Participants had a diagnosis of stage M0 colorectal cancer and were scheduled for elective curative tumor removal surgery. Eligibility criteria required the presence of iron deficiency anemia, defined as hemoglobin levels below 7.5 mmol/l (12 g/dl) for women and below 8 mmol/l (13 g/dl) for men, along with a transferrin saturation of less than 20%	NR	Ferric carboxymaltose.	1000–2000 mg. Severe anemia patients (hemoglobin ≤10 g/dl) received a dose of 1500 mg if their weight was 35–70 kg and 2000 mg if it was more than 70 kg. Patients with mild anemia (hemoglobin >10 g/dl) received a dose of 1000 mg if their weight was 35–70 kg and 1500 mg if it was more than 70 kg	Oral ferrous fumarate	Three tablets of 200 mg	Achieving normal hemoglobin levels before surgery was uncommon with both treatment approaches. Intravenous iron treatment showed significant improvement in hemoglobin levels at all other time points after treatment. Only intravenous iron replenished iron stores

IVI, intravenous iron; NA, not applicable; NR, not reported.

**Table 2:** Baseline characteristics of enrolled patients in each included study.

Study ID	Groups	Number of patients n (%)	Age	Males *n* (%)	BMI, kg/m^2^	Diabetes *n* (%)	HTN *n* (%)	Heart disease *n* (%)	Previous stroke *n* (%)	Iron treatment at diagnosis (%)		Tumor size, cm	Baseline Hb	Baseline Ferritin	Baseline TSAT (%)
										Oral	IVI				
Edwards *et al*., 2009^32^	IV Iron	34 (56.7%)	67 (median)	22 (64.7%)	26.6	N/A	N/A	N/A	N/A	N/A	N/A	N/A	11.7±1.4 (g/dl)	100.5 (ng/ml)	11±12.4
	Control	26 (43.3%)	70 (median)	17 (65.4%)	26.3	N/A	N/A	N/A	N/A	N/A	N/A	N/A	11.8±2.1 (g/dl)	51.5 (ng/ml)	14±11.2
Calleja *et al*., 2016^34^	IV Iron	111 (41.7%)	72.9±11.1	64 (57.3%)	27.7±5.7	32 (29.20%)	62 (56%)	25 (22.60%)	N/A	17 (15.30%)	111 (100%)	N/A	9.6±1.4 (g/dl)	39.6±62.9 (ng/ml)	8.0±5.9
	Control	155 (58.3%)	70.8±10.3	86 (55.8%)	28.2±4.9	55 (35.70%)	87 (56.2%)	30 (19.10%)	N/A	155 (100%)	0 (0%)	N/A	10.0±1.2 (g/dl)	20.0±20.8 (ng/ml)	7.6±4.9
Keeler *et al*., 2017^15^	IV Iron	55 (47.4%)	73.2±8.5	35 (63.6%)	N/A	N/A	N/A	N/A	N/A	25 (45.5%)	N/A	4.1 [3.4–5.5]	N/A	N/A	N/A
	Control	61 (52.6%)	74.4±9.7	37 (60.7%)	N/A	N/A	N/A	N/A	N/A	30 (49.2%)	N/A	4.5 [3.5–6]	N/A	N/A	N/A
Laso-Morales *et al*., 2017^20^	IV Iron	232 (72%)	71±11	135 (58.2%)	28.0±5.0	53 (23%)	121 (52%)	33 (14%)	10 (4%)	N/A	N/A	N/A	10.8±1.5 (g/dl)	52±95 (ng/ml)	9±6
	Control	90 (28%)	69±15	45 (50%)	27.0±5.0	24 (27%)	48 (53%)	19 (21%)	3 (3%)	N/A	N/A	N/A	12.0±0.9 (g/dl)	78±121 (ng/ml)	14±9
Wilson *et al*., 2018^22^	IV Iron	102 (31.9%)	74±9.8	54 (52.9%)	N/A	N/A	N/A	N/A	N/A	N/A	N/A	N/A	9.93±1.81	N/A	N/A
	Control	218 (68.1%)	73.1±10.4	120 (55%)	N/A	N/A	N/A	N/A	N/A	N/A	N/A	N/A	10.79±1.44	N/A	N/A
Kam *et al*., 2020^31^	IV Iron	38 (38%)	70.5 (45–85)	19 (50%)	N/A	9 (23.7%)	21 (55.3)	2 (5.3%)	2 (5.3%)	12 (31.6%)	N/A	N/A	8.43±1.15 (g/dl)	N/A	N/A
	Control	62 (62%)	69 (43–88)	31 (50%)	N/A	18 (29%)	37 (59.7%)	7 (11.3%)	6 (9.7%)	18 (29%)	N/A	N/A	8.79±1.07 (g/dl)	N/A	N/A
Dickson *et al*., 2020^33^	IV IRON	54 (49.1%)	74.1±8.8	35 (65%)	N/A	N/A	N/A	N/A	N/A	25 (46%)	N/A	3.98 [2.19]	9.6±1.3 (g/dl)	N/A	N/A
	Control	56 (50.9%)	75.2±11.1	34 (61%)	N/A	N/A	N/A	N/A	N/A	28 (50%)	N/A	4.35 [3.48]	9.8±1.1 (g/dl)	N/A	N/A
Kangaspunta *et al*., 2022^30^	IV Iron	180 (56.6%)	73.6±9.86	84 (46.7%)	25.8±4.48	N/A	N/A	N/A	N/A	N/A	N/A	N/A	9.9±1.55 (g/dl)	N/A	N/A
	Control	138 (43.4%)	76±8.84	72 (52.2%)	26.5±5.16	N/A	N/A	N/A	N/A	N/A	N/A	N/A	N/A	N/A	N/A
Ploug *et al*., 2022^29^	IV Iron	122 (71.8%)	75.7±9.0	68 (55.7%)	26.1±3.9	N/A	N/A	N/A	N/A	N/A	N/A	N/A	9.5±2.0 (g/dl)	13.00 [8–22] (μg/l)	N/A
	Control	48 (28.2%)	73.5±14.5	22 (45.8%)	25.3±4.4	N/A	N/A	N/A	N/A	N/A	N/A	N/A	9.7±3.4 (g/dl)	14.50 [9–26] (μg/l)	N/A
Fung *et al*., 2022^35^	IV IRON	20 (50%)	68.4±6.8	15 (75%)	23.1±3.8	9 (45%)	11 (55%)	2 (10%)	1 (5%)	N/A	N/A	4.5 [3.0–6.8]	10.8±1.4 (g/dl)	20.9 [12.6–27.5] (μg/l)	9.5 [5.0–12.8]
	Control	20 (50%)	69.8±12.6	9 (45%)	22.4±4.6	7 (35%)	11 (55%)	1 (5%)	1 (5%)	N/A	N/A	3.5 [3.0-4.0]	10.5±1.4 (g/dl)	11.6 [6.6–19.7] (μg/l)	10.0 [6.3–15.0]
Talboom *et al*., 2023^28^	IV Iron	96 (47.5%)	71.3±12	49 (51%)	27±6;	N/A	N/A	19 (20%)	N/A	1 (1%)	95 (99%)	N/A	10.5 (g/dl)	63 (μg/l)	N/A
	Control	106 (53.5%)	70.7±15	56 (53%)	26±5	N/A	N/A	23 (22%)	N/A	104 (98%)	2 (2%)	N/A	10.3 (g/dl)	64 (μg/l)	N/A

Data are expressed as median (range), mean±SD or Median [IQR value].

Hb, hemoglobin; HTN, hypertension; IVI, intravenous Iron; N/A, not available; TSAT, transferrin saturation.

### Quality assessment

Upon utilizing the Cochrane Rob2 tool to assess the risk of bias in the included randomized controlled trials (RCTs), it was observed that Keeler *et al*.^15^ and Fung *et al*.^35^ exhibited an overall low risk, whereas Dickson *et al*.^33^and Talboom *et al*.^28^ presented an overall high risk. Edwards *et al*.^32^raised some concerns based on the five domains of the RoB-2 tool. Notably, the studies by Dickson *et al*.^33^and Talboom *et al*.^28^were designated as having an overall high risk of bias due to inadequacy in their randomization processes.

Turning to the observational studies, the majority demonstrated good quality scores according to the NOS tool. However, Wilson *et al*.^22^ and Kam *et al*.^31^ received fair scores in terms of quality. Additional details regarding the quality assessment can be found in Supplementary Figures 1 and 2 (Supplemental Digital Content 3, http://links.lww.com/MS9/A639), as well as Supplementary Table 2 (Supplemental Digital Content 3, http://links.lww.com/MS9/A639).

### Publication bias

Assessing publication bias in our review was not feasible due to the insufficient reporting of outcomes by at least 10 studies.

### Efficacy outcomes

#### Change from the baseline in hemoglobin

Our pooled analysis demonstrated a statistically significant increase in Hb levels following IVI supplementation compared to standard care at various time points: preoperative (MD=1.17, 95% CI [0.95–1.40], *P*<0.01), postoperative day one (MD=1.32, 95% CI [0.89–1.76], *P*<0.01), hospital discharge (MD=0.76, 95% CI [0.28–1.24], *P*=0.002), and 30 days postoperative (MD=1.57, 95% CI [1.27–1.87], *P*<0.01).

**Figure 2:**
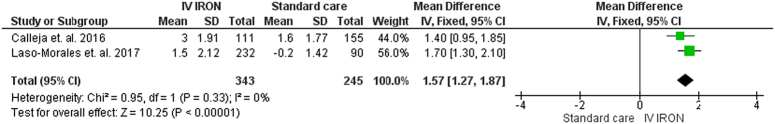
Forrest plot demonstrates change from the baseline in Hb level at 30 days postoperative.

**Figure 3:**
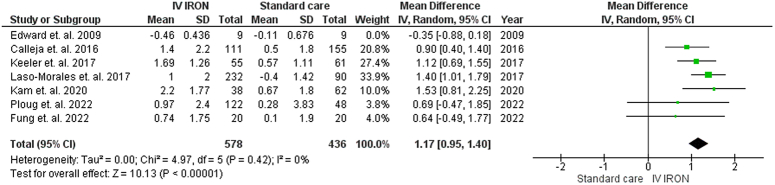
Forrest plot demonstrating the change from the baseline in Hb level at preoperative period.

Although the pooled studies at 30 days postoperative period were homogenous (*P*=0.33, *I* ^2^=0%) (Fig. 2), heterogeneity was observed in the pooled studies at preoperative (*P*<0.0001, *I* ^2^=81%), postoperative day one (*P*=0.05, *I* ^2^=66%), and hospital discharge (*P*=0.03, *I* ^2^=66%) periods. Heterogeneity during the preoperative period was resolved by excluding Edward *et al*. 2009^32^ (*P*=0.42, *I* ^2^=0%) (Fig. 3). Similarly, during the postoperative day one period, heterogeneity was resolved by excluding Edward *et al*. 2009^32^ (*P*=0.44, *I* ^2^=0%) (Supplementary File Figure 3, Supplemental Digital Content 3, http://links.lww.com/MS9/A639). Moreover, during the hospital discharge period, heterogeneity was resolved by excluding Laso-Morales *et al*. 2017^20^ (*P*=0.33, *I* ^2^=9%) (Supplementary File Figure 4, Supplemental Digital Content 3, http://links.lww.com/MS9/A639).

#### Need for RBC transfusions

Our pooled analysis demonstrated a statistically significant decrease in the overall need for RBC transfusions with IVI supplementation compared to standard care (RR=0.39, 95% CI [0.27–0.56], *P*<0.01). However, when examining specific time points, our analysis demonstrated a nonstatistically significant difference between IVI and standard care during the preoperative period (RR=0.93, 95% CI [0.56–1.53], *P*=0.76) and intraoperative period (RR=0.84, 95% CI [0.58–1.22], *P*=0.36). Interestingly, the postoperative period showed a statistically significant decrease in the need for RBC transfusions with IVI supplementation compared to standard care (RR=0.69, 95% CI [0.52–0.92], *P*=0.01).

**Figure 4:**
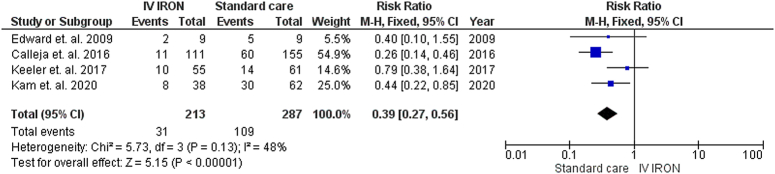
Forrest plot illustrating the overall need for RBC transfusion.

Low heterogeneity was observed in the overall need for RBC transfusions (*P*=0.13, *I* ^2^=48%) (Fig. 4), intraoperative need for RBC transfusions (*P*=0.33, *I* ^2^=12%) (Supplementary File Figure 5, Supplemental Digital Content 3, http://links.lww.com/MS9/A639), and postoperative need for RBC transfusions (*P*=0.20, *I* ^2^=32%) (Supplementary File Figure 6, Supplemental Digital Content 3, http://links.lww.com/MS9/A639). However, the preoperative need for RBC transfusions demonstrated high heterogeneity(*P*=0.02), *I*²=61%), which was resolved by excluding Kam *et al*. 2020^31^ (*P*=0.21, *I* ^2^=32%) (Supplementary File Figure 7, Supplemental Digital Content 3, http://links.lww.com/MS9/A639).

#### Overall mean number of RBC units transfused

**Figure 5:**
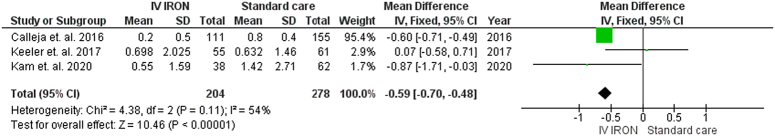
Forrest plot depicting the overall mean units of RBC transfused.

Our pooled analysis demonstrated a statistically significant decrease in the overall mean number of RBC units transfused with IVI supplementation compared to standard care (MD=−0.59, 95% CI [−0.70 to −0.48], *P*<0.01). The results demonstrated low heterogeneity (*P*=0.11, *I* ^2^=54%) (Fig. 5).

### Safety outcomes

#### Total complications

**Figure 6:**
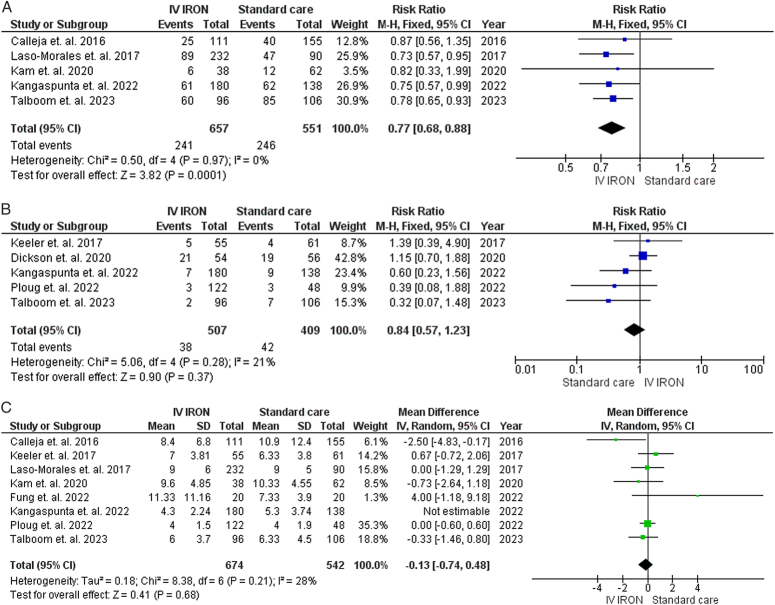
A: Forrest plot showing total complications, B: Forrest plot showing total death, C: Forrest plot showing hospital stay.

Our pooled analysis demonstrated a statistically significant decrease in total complications with IVI supplementation compared to standard care (RR=0.77, 95% CI [0.68–0.88], *P*<0.01). The results demonstrated low heterogeneity (*P*=0.97, *I* ^2^=0%) (Fig. 6A).

#### Total death

Our pooled analysis demonstrated a nonstatistically significant difference between IVI supplementation and standard care (RR=0.84, 95% CI [0.57–1.23], *P*=0.37). The results demonstrated low heterogeneity (*P*=0.28, *I* ^2^=21%) (Fig. 6B).

#### Hospital stay

Our pooled analysis demonstrated a nonstatistically significant difference between IVI supplementation and standard care (RR=−0.13, 95% CI [−0.74 to 0.48], *P*=0.68). The results demonstrated high heterogeneity (*P*=0.07, *I* ^2^=46%) resolved by excluding Kangaspunta *et al*. 2022^30^ (*P*=0.21, *I* ^2^=28%) (Fig. 6C).

#### Total infection

Our pooled analysis demonstrated a nonstatistically significant difference between IVI supplementation and standard care (RR=1.06, 95% CI [0.72–1.55], *P*=0.77). The results demonstrated high heterogeneity (*P*=0.05, *I* ^2^=56%) resolved by excluding Laso-Morales *et al*. 2017^20^ (*P*=0.26, *I* ^2^=25%) (Supplementary File Figure 8, Supplemental Digital Content 3, http://links.lww.com/MS9/A639).

#### Wound infection

Our pooled analysis demonstrated a nonstatistically significant difference between IVI supplementation and standard care (RR=0.58, 95% CI [0.29–1.19], *P*=0.14). The results demonstrated low heterogeneity (*P*=0.91, *I* ^2^=0%) (Supplementary File Figure 9, Supplemental Digital Content 3, http://links.lww.com/MS9/A639).

#### Wound dehiscence

Our pooled analysis demonstrated a nonstatistically significant decrease in wound dehiscence with IVI supplementation compared to standard care (RR=0.69, 95% CI [0.49–0.98], *P*=0.04). The results demonstrated low heterogeneity (*P*=0.97, *I* ^2^=0%) (Supplementary File Figure 10, Supplemental Digital Content 3, http://links.lww.com/MS9/A639).

#### Paralytic ileus

Our pooled analysis demonstrated a nonstatistically significant difference between IVI supplementation and standard care (RR=0.89, 95% CI [0.60–1.31], *P*=0.55). The results demonstrated low heterogeneity (*P*=0.42, *I* ^2^=0%) (Supplementary file figure 11, Supplemental Digital Content 3, http://links.lww.com/MS9/A639).

#### Anastomotic leakage

Our pooled analysis demonstrated a nonstatistically significant difference between IVI supplementation and standard care (RR=0.90, 95% CI [0.32–2.53], *P*=0.84). The results demonstrated low heterogeneity (*P*=0.93, *I* ^2^=0%) (Supplementary file figure 12, Supplemental Digital Content 3, http://links.lww.com/MS9/A639).

### Survival outcome

#### Overall survival

Our pooled analysis demonstrated a nonstatistically significant difference between IVI supplementation and standard care in overall survival at 1 year (RR=0.98, 95% CI [0.92–1.05], *P*=0.63), 3 years (RR=0.94, 95% CI [0.83–1.07], *P*=0.36), and 5 years (RR=0.91, 95% CI [0.77–1.08], *P*=0.27). Low heterogeneity was observed in overall survival at 1 year (*P*=0.76, *I* ^2^=0%), 3 years (*P*=0.72, *I* ^2^=0%), and 5 years (*P*=0.95, *I* ^2^=0%) (Supplementary File Figures 13,14, and 15, respectively, Supplemental Digital Content 3, http://links.lww.com/MS9/A639).

#### Disease-free survival

Our pooled analysis demonstrated a nonstatistically significant difference between IVI supplementation and standard care in disease-free survival at 1 year (RR=1.02, 95% CI [0.94–1.11], *P*=0.62), 3 years (RR=1.09, 95% CI [0.96–1.25], *P*=0.18), and 5 years (RR=1.02, 95% CI [0.88–1.18], *P*=0.77).

Low heterogeneity was observed in disease-free survival at 1 year (*P*=0.38, *I* ^2^=0%), 3 years (*P*=0.58, *I* ^2^=0%), and 5 years (*P*=0.33, *I* ^2^=0%) (Supplementary File Figures 16, 17, and 18, respectively, Supplemental Digital Content 3, http://links.lww.com/MS9/A639).

## Discussion

### Summary of the findings

In our meta-analysis, IVI demonstrated a consistent increase in Hb levels at various time points—preoperative, postoperative day one, hospital discharge, and 30 days postoperative—when compared to standard care. Additionally, IVI supplementation resulted in a notable decrease in the overall need for RBCT in comparison to standard care. However, this reduction was not consistently observed across all time points. IVI significantly reduced the need for RBCT, specifically in the postoperative period, with no significant difference during the preoperative and intraoperative periods.

Moreover, IVI supplementation demonstrated significantly lower rates of total complications and wound dehiscence compared to standard care. Conversely, there were no statistically significant differences between IVI supplementation and standard care in total death, hospital stay, total infection, wound infection, paralytic ileus, anastomotic leakage, overall survival, and disease-free survival.

### Explanation of the findings

Preoperative anemia in CRC commonly results from chronic bleeding, the consequences of neoadjuvant chemotherapy or radiotherapy, and nutritional deficiencies. The development of preoperative anemia in CRC is intricately linked to inflammatory mediators such as TNF-α, IFN-γ, and various interleukins. These mediators contribute to anemia through mechanisms like dyserythropoiesis, inadequate erythropoietin response, and disrupted iron homeostasis^36–38^. Hepcidin, influenced by IL-6, also plays a pivotal role in regulating iron absorption and contributing to functional iron deficiency (FID)^36–39^. FID may progress to absolute iron deficiency (FID + ID) with sustained decreased iron absorption or chronic blood loss^39^.

Understanding hepcidin levels is crucial in distinguishing between FID and FID + ID, guiding appropriate therapy choices, such as IVI for those with low hepcidin levels. Studies demonstrate the effectiveness of IVI, particularly in chemotherapy-induced anemia with low hepcidin levels. Hepcidin levels also aid in predicting nonresponsiveness to oral iron therapy in ID anemia patients^39–41^.

Ward *et al*.^42^ investigated hepcidin in CRC tissue, revealing its correlation with ferroportin inhibition. The study emphasized that CRC-associated anemia, more likely to be ID or FID + ID, responds more favorably to intravenous than oral iron. In cases of FID + ID, comprehensive testing is essential to exclude other causes, involving B12, lactate dehydrogenase, serum creatinine, and red cell folate for malabsorption or severe malnutrition.

After CRC surgery, there is a risk of severe postoperative anemia due to blood loss and inflammation induced by surgery, especially in those with preoperative anemia^43^. The swift elevation of Hb levels through allogeneic RBCT is accompanied by adverse outcomes, including increased risks of cancer recurrence and mortality^43^. To mitigate these risks, the use of RBCT should be restricted to severe cases with immediate symptoms.

The objective of preoperative anemia therapy is to achieve normal Hb levels, aligning with WHO criteria. Given the moderate-to-high blood loss in CRC resections, targeting a Hb of 13 g/dl for both genders is advisable to minimize the risk of transfusion^43^. The delivery of perioperative iron supplementation can be achieved through various means, including oral or intravenous methods. While some studies explored the efficacy of preoperative oral iron, results consistently favor IVI for better outcomes^15^. Moreover, Intraoperative IVI iron further supports postoperative hemoglobin by enhancing hematopoiesis and iron availability, leading to faster recovery, and reducing the need for allogeneic transfusions especially RBCT. This makes IVI a valuable strategy for managing blood loss and improving recovery after surgery^44,45^.

Doubts about the effectiveness of oral iron have emerged due to challenges in gastrointestinal absorption or the presence of concurrent chronic medical conditions^46^. Thus, IVI is put forth as a more efficient choice, especially in situations involving acute or chronic inflammatory processes where the inhibitory effects of hepcidin on gastrointestinal absorption may impede oral iron’s efficacy^47^. Despite concerns about potential adverse effects such as anaphylaxis and a theoretical risk of infection associated with IVI^48^, recent studies suggest that newer preparations have demonstrated safety concerning anaphylaxis and have not been associated with increased infection rates^49^. Therefore, IVI is recommended for correcting ID anemia before CRC surgery, preferably administered 2 to 4 weeks before the scheduled procedure^43^.

According to our meta-analysis, there are a number of reasons why the need for red blood cell transfusion (RBCT) during the preoperative phase did not reach statistical significance, even if intravenous iron (IVI) supplementation raised hemoglobin (Hb) levels. IVI may increase preoperative Hb levels, although they may not surpass the transfusion cut-off, which is usually based on certain Hb thresholds^50,51^. Furthermore, each patient reacts differently to IVI; some experience greater increases in hemoglobin than others. Clinical decision-making considers factors such as patient tolerance, comorbidities, and anemia symptoms in addition to Hb levels, which may lessen the effect of IVI-induced Hb rises^52^. The ability to identify notable variations in RBCT needs may be impacted by additional contributing factors, including patient characteristics, study design variations, and population size.

The lack of significance in RBCT during the intraoperative phase may be the result of inflammation brought on by surgery, which has an impact on red blood cell formation and iron metabolism^53^. Additionally, hemodilution may result from the significant fluid supply during surgery, making it more difficult to evaluate Hb levels and make RBCT judgments. IVI helps heart failure patients with iron deficient anemia, according to prior meta-analyses; however, these advantages might not always be immediately apparent in surgical settings^54,55^.

Furthermore, IVI supplementation significantly reduced overall complications in our meta-analysis of colorectal cancer resections. Higher surgical complications, longer hospital stays, and a lower disease-free survival are all closely linked to preoperative anemia, particularly when the hematocrit is less than 30%^43^. An increased risk of postoperative infections is also associated with low preoperative serum ferritin levels^56^. While Fjørtoft *et al*.^57^ observed worse survival rates with preoperative anemia, Zago *et al*.^58^ discovered that higher erythrocyte protoporphyrin, a measure of iron shortage, was associated with more problems. In contrast to normal therapy, IVI supplementation had no discernible effect on hospital stays, infection rates, or survival outcomes.

### Agreements and disagreements with previous studies

Our analysis encompasses data from 11 studies, featuring a substantial pooled sample size of 2024 patients. In comparison, the meta-analysis conducted by Hallet *et al*.^21^ encompasses four studies with a pooled sample size of 325 patients. Their study specifically focuses on interventions involving perioperative iron, administered either intravenously or orally, compared to no intervention, in individuals undergoing gastrointestinal surgery for benign or malignant diseases. On the other hand, the meta-analysis conducted by Yang *et al*.^59^ involves six studies, with a sample size of 855 patients. Their investigation is centered on interventions that include oral or IVI supplementation versus placebo or no iron supplementation. This meta-analysis specifically addresses elderly patients undergoing hip or knee surgery.

In the context of the need for RBC transfusions and the mean number of RBC units transfused, our findings differ from those of Hallet *et al*.^21^ and Yang *et al*.^59^. Their results show no statistically significant differences in the need for RBC transfusions and the mean number of RBC units transfused. However, concerning the change in baseline Hb, Yang *et al*.^59^ found a statistically significant difference between iron treatment and no iron treatment at the end of the treatment regimen, aligning with our findings. In terms of hospital stay, our study aligns with Hallet *et al*.^21^ and Yang *et al*.^59^, both of which found no significant difference between the intervention and control groups.

Regarding mortality and infection rates, our study aligns with Yang *et al*.^59^, which found no significant difference between the intervention and control groups. However, concerning complications, our study contrasts with Yang *et al*.^59^, in which we found a significant difference between IVI and standard care.

Taking in consideration that, there is a distinction between CRC surgery and orthopedic surgery. In CRC patients, anemia is chronic and primarily linked to the underlying disease rather than surgical blood loss. This fundamental difference suggests that the impact of iron intervention may vary in these distinct contexts^60–64^.

### Strength points and limitations

Our study stands as the first comprehensive meta-analysis comparing IVI supplementation against standard care, encompassing no iron or oral iron, specifically in CRC patients with ID anemia. Our inclusion comprised 11 studies, with 5 being RCTs and 6 being observational studies, totaling 2024 patients. The study thoroughly examined various outcomes, including the change from baseline in Hb, the need for RBC transfusions, the overall mean number of RBC units transfused, overall survival, and disease-free survival. Additionally, safety measures were comprehensively assessed, encompassing total complications, total death, hospital stay, total infection, wound infection, anastomotic leakage, wound dehiscence, and paralytic ileus. Additionally, our analysis demonstrated homogeneity in most outcomes.

Addressing the limitations of our analysis, it is important to note that our study exclusively included English-language studies. The majority of the studies reviewed were retrospective, potentially introducing an increased risk of bias, especially in terms of patient selection. Our analysis did not encompass outcomes such as quality of life, changes in baseline ferritin and transferrin saturation, and the prevalence of preoperative, intraoperative, and postoperative anemia, as these data were not reported in the included studies. Furthermore, certain analyses were conducted based on data from only two studies, which may have implications for the generalizability of our findings. Furthermore, inherent constraints hindered our ability to perform a subgroup analysis comparing IVI to oral iron and IVI to no iron. Lastly, due to the limited availability of data and studies, we could not perform an analysis to explore the relationship between anemia severity and improvement of outcomes.

### Implications and recommendations

The implications of our findings suggest that IVI supplementation holds promise in optimizing outcomes for CRC patients with ID anemia. The sustained effectiveness of IVI supplementation in increasing Hb levels at various time points demonstrates its reliability compared to standard care. Importantly, our findings reveal a significant reduction in the overall need for RBCT with IVI supplementation, particularly during the postoperative period, suggesting the potential clinical benefits of IVI. This not only contributes to better Hb management but also aligns with the broader goal of minimizing the reliance on RBCT. Furthermore, the significantly lower rates of total complications and wound dehiscence associated with IVI supplementation highlight its potential role in reducing postoperative complications and better resource utilization.

While certain time points, such as preoperative and intraoperative, did not show statistically significant differences in the need for RBCT, the overall trends in Hb improvement, decreased overall need for RBCT, and reduced complications suggest that IVI supplementation could be a valuable adjunct in the management of CRC patients undergoing surgery. These findings provide clinicians with valuable insights for considering IVI as part of a comprehensive strategy to enhance outcomes in this patient population.

Thus, we call for more RCTs to be conducted on such an important topic taking into consideration assessing more patient-oriented and clinically relevant outcomes such as change in quality of life, change in baseline ferritin and transferrin saturation, and the prevalence of preoperative, intraoperative, and postoperative anemia. Moreover, to further separate control groups into either oral iron or no iron to investigate the efficacy of each as a separate entity.

## Conclusion

In conclusion, our meta-analysis emphasizes the effectiveness of IVI supplementation in increasing Hb levels and reducing the overall need for RCBT, especially in the postoperative period. Although specific time points like preoperative and intraoperative did not show significant differences, overall trends suggest a potential clinical benefit of IVI. Additionally, IVI supplementation was linked to lower rates of complications and wound dehiscence, indicating effective postoperative outcomes. Further research addressing limitations and exploring additional outcomes is warranted.

## Ethical approval

Efficacy and safety of preoperative intravenous iron versus standard care in colorectal cancer patients with iron deficiency anemia, a systematic review and meta-analysis.

## Consent

Informed consent was not required for this systematic review.

## Source of funding

All author(s) received no financial support for the research, authorship, and/or publication of this article.

## Author contribution

P.S.: led the team, overseeing the search strategy’s development and execution, ensuring idea validity, and addressing conflicts during screening and quality evaluation; M.S.A.: contributed to analysis and authored the introduction and discussion; B.A.-Q.: handled the results section and tables; N.S.: engaged in screening, data extraction, and methods writing; M.E.-S.: assessed quality and contributed to the introduction; N.N. and N.A.: handled data extraction for baseline and outcomes; M.T.O.: participated in screening and data extraction; A.M.: contributed to quality assessment; A.K.A.: supervised and conducted a thorough peer-review. All authors actively participated in manuscript review and collectively endorsing the final version.

## Conflicts of interest disclosure

All authors declare no conflict of interest.

## Research registration unique identifying number (UIN)

PROSPERO under registration number [CRD42023481770].

## Guarantor

Not applicable.

## Data availability statement

All data generated or analyzed during this study are included in this published article or the data repositories listed in the references.

## Provenance and peer review

Not commissioned, externally peer-reviewed.

## Supplementary Material
