# Supplementary material for: Efficacy and safety of preoperative intravenous iron versus standard care in colorectal cancer patients with iron deficiency anemia: a systematic review and meta-analysis
Source: Ann Med Surg (Lond). 2024 Nov 11;86(12):7105–19. doi: 10.1097/MS9.0000000000002727 (PMC11623905; doi:10.1097/MS9.0000000000002727)
Supplement: Supplementary file 3 [file ms9-86-7105-s003.docx]

**Title: Efficacy and safety of** **Preoperative Intravenous Iron versus standard care in colorectal cancer patients with Iron Deficiency Anemia - Systematic Review and Meta-Analysis**

**Supplementary figures:**

**Supplementary Figure 1**. Risk of bias summary for randomized controlled trials using ROB2.


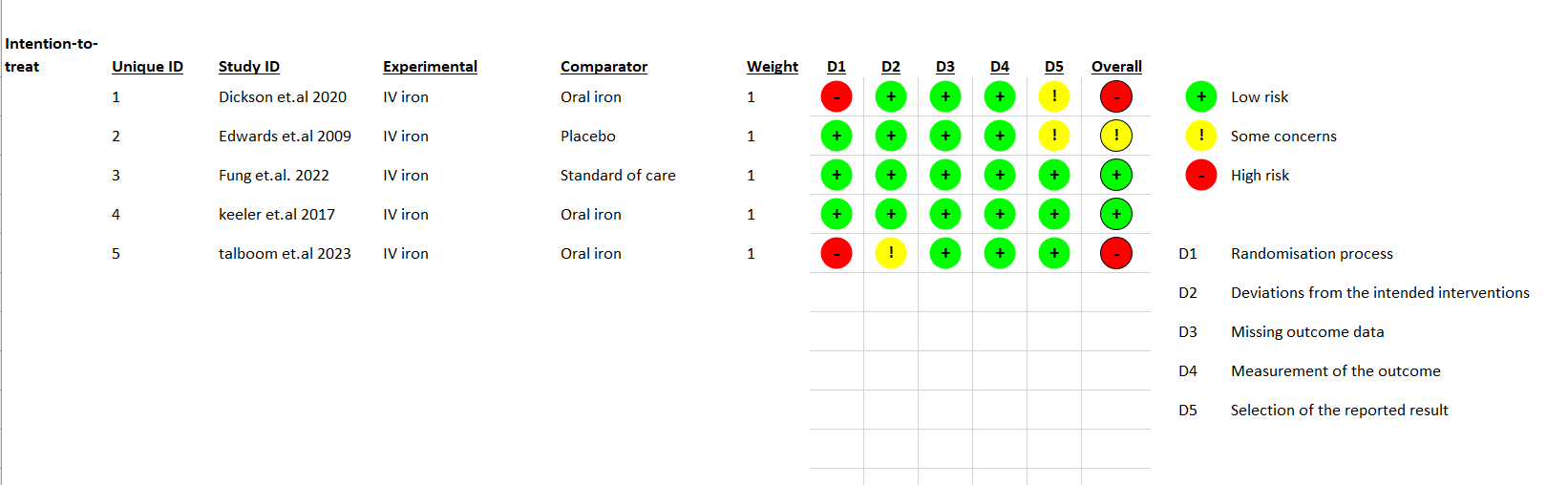


**Supplementary Figure 2**. Risk of bias graph for randomized controlled trials using ROB2.


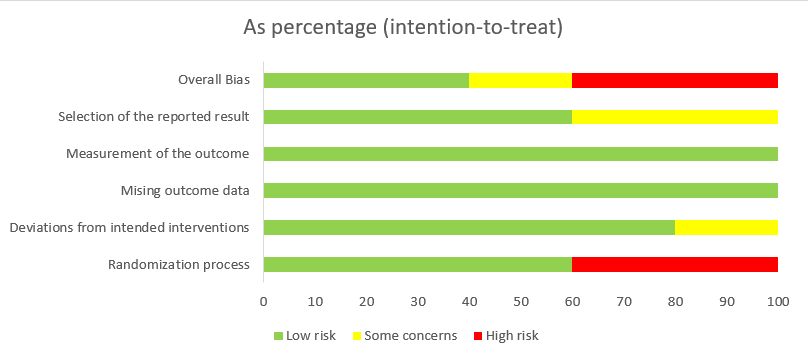


**Supplementary Figure 3.** Forrest plot demonstrates change from the baseline in Hb level at postoperative day one.


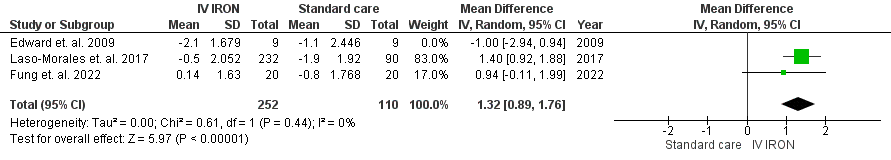


**Supplementary Figure 4.** Forrest plot demonstrates change from the baseline in Hb level at Hospital discharge.


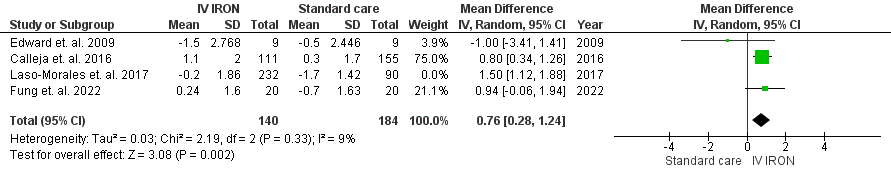


**Supplementary Figure 5.** Forrest plot demonstrates intraoperative need for RBC transfusions.


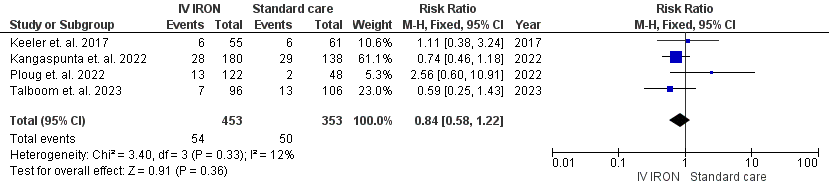


**Supplementary Figure 6.** Forrest plot demonstrates postoperative need for RBC transfusions.


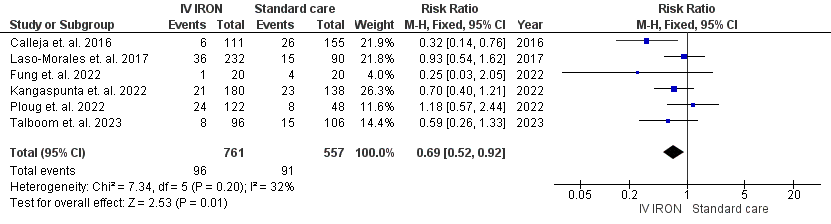


**Supplementary Figure 7.** Forrest plot demonstrates preoperative need for RBC transfusions.


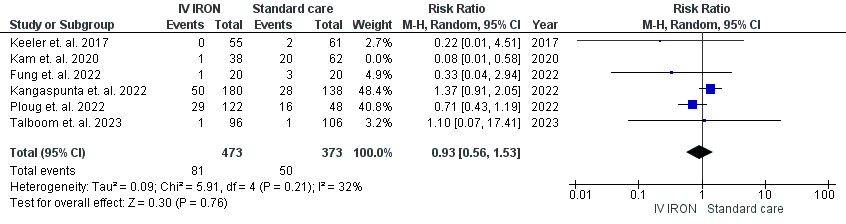


**Supplementary Figure 8.** Forrest plot demonstrates total Infections.


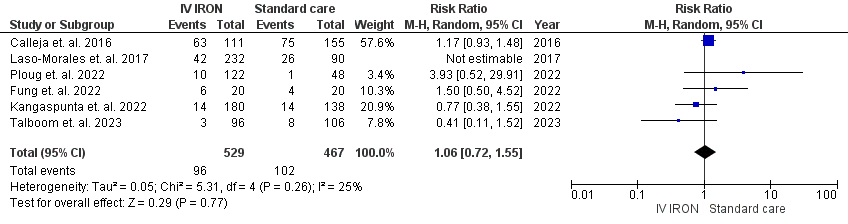


**Supplementary Figure 9.** Forrest plot demonstrates wound Infections.


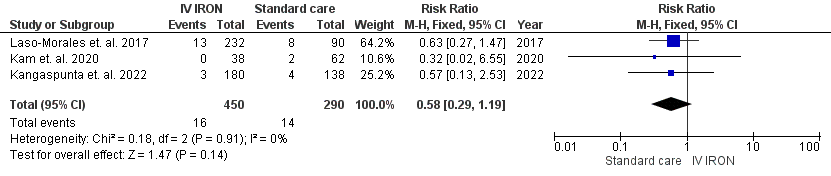


**Supplementary Figure 10.** Forrest plot demonstrates wound dehiscence.


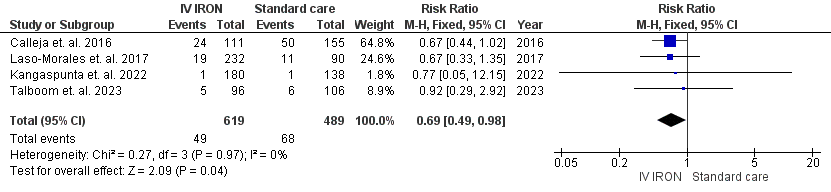


**Supplementary figure 11.** Forrest plot demonstrates paralytic ileus.


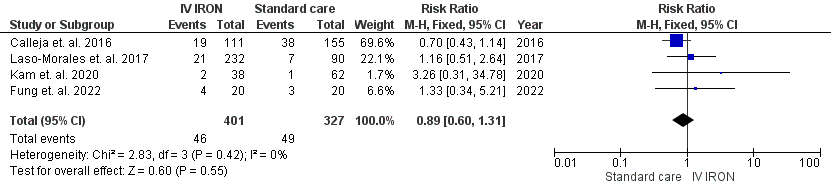


**Supplementary figure 12.** Forrest plot demonstrates anastomotic leakage.


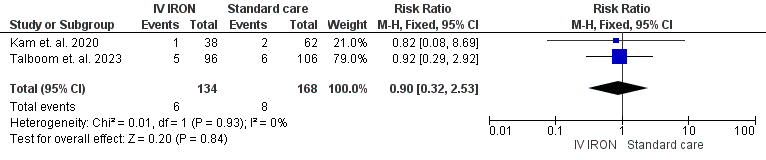


**Supplementary Figure 13.** Forrest plot demonstrates overall survival at 1 year.


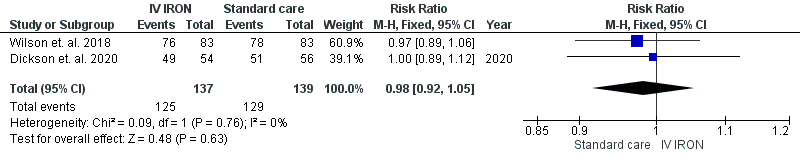


**Supplementary Figure 14.** Forrest plot demonstrates overall survival at 3 years.


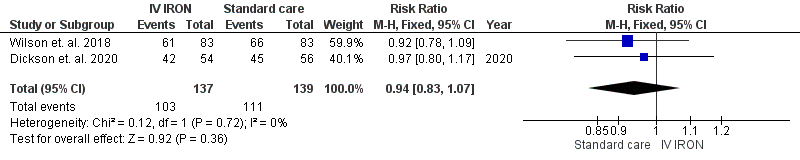


**Supplementary figure 15.** Forrest plot demonstrates overall survival at 5 years.


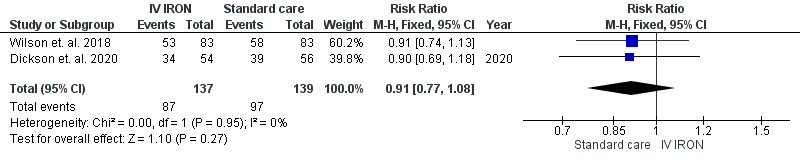


**Supplementary Figure 16.** Forrest plot demonstrates disease-free survival at 1 year.


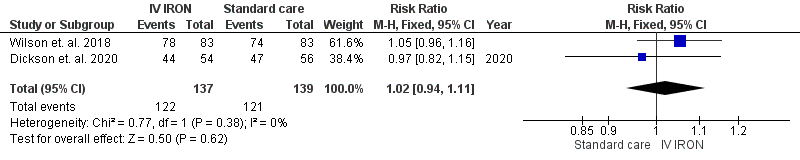


**Supplementary Figure 17.** Forrest plot demonstrates disease-free survival at 3 years.


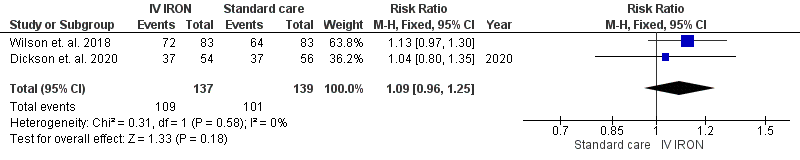


**Supplementary Figure 18.** Forrest plot demonstrates disease-free survival at 5 years.


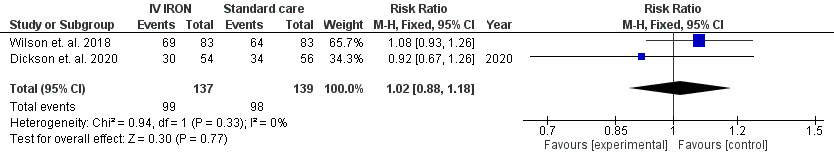


**Supplementary tables:**

**Supplementary table 1**: Search strategy application through different data-bases

| Database | Search strategy | N of results |
| --- | --- | --- |
| **PubMed** | (Colorectal Neoplasm OR Colorectal Tumors OR Colorectal Tumor OR Colorectal Cancer OR Colorectal Cancers OR Colorectal Carcinoma OR Colorectal Carcinomas) AND (Iron Deficiency anemia*) AND (Ferridextran OR ferric OR ferrous OR iron) | **307** |
| **Cochrane** | (Colorectal Neoplasm OR Colorectal Tumors OR Colorectal Tumor OR Colorectal Cancer OR Colorectal Cancers OR Colorectal Carcinoma OR Colorectal Carcinomas) AND (Iron Deficiency anemia*) AND (Ferridextran OR ferric OR ferrous OR iron) | **44** |
| **Web of Science** | (Colorectal Neoplasm OR Colorectal Tumors OR Colorectal Tumor OR Colorectal Cancer OR Colorectal Cancers OR Colorectal Carcinoma OR Colorectal Carcinomas) AND (Iron Deficiency anemia*) AND (Ferridextran OR ferric OR ferrous OR iron) | **299** |
| **Scopus** | (“Colorectal Neoplasm” OR “Colorectal Tumors” OR ”Colorectal Tumor” OR “Colorectal Cancer” OR “Colorectal Cancers” OR” Colorectal Carcinoma” OR “Colorectal Carcinomas”) AND (“Iron Deficiency anemia*”) AND (Ferridextran OR ferric OR ferrous OR iron) | **559** |

**Supplementary table 2**: Methodological quality assessment of the included 6 studies, based on the NOS for assessing the quality of observational studies.

1. Cohort studies (n=6)

| **Study** | **Selection** | | | | **Comparability** | **Outcome** | | | **Total Score** |
| --- | --- | --- | --- | --- | --- | --- | --- | --- | --- |
|  | **Representativeness of the exposed cohort** | **Selection of the non-exposed cohort** | **Ascertainment of exposure^5^** | **Outcome was not present at start of study^6^** | **Control for 2 important factors^2,3^** | **Assessment of outcome** | **Follow-up long enough** | **Adequacy of follow-up of cohort^7^** |  |
| Calleja et al, 2016 ^34^ | somewhat representitive of the average* | drawn from the same community* | secure record* | yes* | study control for intraoperative blood losses and age** | independent blinded assessment * | yes 2-3 weeks * | complete follow up* | (9) Good |
| Laso‐Morales et al, 2017 ^20^ | selected group of useres | drawn from the same community* | secure record* | yes* | study control poor for size of groups compared to each other but good for comorbidities * | record linkage * | no not stated | complete follow up* | (6) Good |
| Wilson et al, 2018 ^22^ | selected group of useres | drawn from different source | secure record* | yes* | study control poor for size of groups compared to each other but good for ASA scores* | record linkage * | yes 2-3 weeks * | complete follow up* | (6) Fair |
| Kam et al, 2020 ^31^ | selected group of useres | no description of the derivation of the non exposed cohort | secure record* | yes* | study control good for of both groups but poor ASA scores (not mentioned) * | record linkage * | yes 2-3 weeks * | complete follow up* | (6) Fair |
| Kangaspunta, 2022 ^30^ | selected group of useres | drawn from the same community* | secure record* | yes* | study control for study control for age and ASA score** | record linkage * | yes 2-3 weeks * | complete follow up* | (8) Good |
| Ploug et al, 2022 ^29^ | selected group of useres | drawn from the same community* | secure record* | yes* | study control poor for size of groups compared to each other but good for ASA scores* | record linkage * | no not stated | complete follow up* | (6) Good |

^1^ If the cases data was obtained from records with mentioning the process to extract information, or reference to primary record, a point was assigned.
^2^ If adjusted for age, a point was assigned.
^3^ If adjusted for drugs (e.g. anti-hypertensives, anti-diabetics etc) or any other additional factors, a point was assigned.
^4^ If information were obtained through national registries or hospital records, a point was assigned.
^4^ If information were obtained from patients and the difference in non-response rate between groups was 20% or less a point was assigned.
^5^ If the exposure data was obtained from prescription database or medical record, a point was assigned.
^6^ If the study design is prospective study, a point was assigned.
^7^ If the completeness of follow-up was 80% or more, a point was assigned.
